# Assessment of foot health and animal welfare: clinical findings in 229 dairy Mediterranean Buffaloes (Bubalus bubalis) affected by foot disorders

**DOI:** 10.1186/s12917-016-0726-4

**Published:** 2016-06-14

**Authors:** Jacopo Guccione, Christian Carcasole, Maher Alsaaod, Luigi D’Andrea, Antonio Di Loria, Angela De Rosa, Paolo Ciaramella, Adrian Steiner

**Affiliations:** Department of Veterinary Medicine and Animal Productions, University of Napoli “Federico II”, Via F. Delpino 1, 80137 Naples, Italy; Veterinary practitioner, Latina District, 04100 Italy; Clinic for Ruminants, Department of Clinical Veterinary Medicine, Vetsuisse-Faculty, University of Berne, 3012 Bern, Switzerland; Department of Health Science, University of Magna Graecia of Catanzaro, 88100 Catanzaro, Italy

**Keywords:** Foot disorders, Claw, Welfare, Lameness, Mediterranean Buffalo, Corkscrew claw, White line disease, Interdigital phlegmona, Digital dermatitis, Interdigital hyperplasia

## Abstract

**Background:**

Lameness represents the third most important health-related cause of economic loss in the dairy industry after fertility and mastitis. Although, dairy Mediterranean Buffaloes (MB) and dairy cows share similar breeding systems predisposing to similar herd problems, published studies exploring its relevance and role in these ruminants are still rare and incomplete. The aims of this study were to describe the clinical findings of foot disorders (FDs) in dairy MB and their influence on animal welfare, determined by assessment of locomotion score (LS), body condition score (BCS) and cleanliness score (CS).

**Results:**

Of 1297 multiparous MB submitted to routine trimming procedures, 229 buffaloes showed at least one FD. The prevalence of buffaloes affected by FDs was 17.7 %, while motility and lameness indexes were 84.1 % (1091/1297) and 15.9 % (206/1297), respectively. Overgrowth was present in 17.0 % (220/1297), corkscrew claw in 15.8 % (205/1297), interdigital phlegmon in 0.9 % (12/1297), white line abscess in 0.8 % (11/1297), digital dermatitis in 0.1 % (1/1297) and interdigital hyperplasia in 0.1 % (1/1297). Simultaneous presence of FDs was recorded in 17.0 % of MB (221/1297): overgrowth and corkscrew claw occurred together in 15.8 % of cases (205/1297), overgrowth and interdigital phlegmon in 0.3 % (4/1297), overgrowth and white line abscess in 0.8 % (11/1297), digital dermatitis and interdigital hyperplasia in 0.1 % (1/1297). The presence of FDs was always associated with lameness (LS > 2), except from 23 MB with simultaneous overgrowth and interdigital phlegmon occurrence. The majority of MB within the under-conditioned group (95.5 %, 43/45) and all those with CS > 2 (122/122) had a locomotion score above the threshold of normality (LS > 2). Furthermore, foot diseases such as interdigital hyperplasia, white line abscess and digital dermatitis or interdigital hyperplasia seemed to occur more frequently associated with decreased BCS and increased CS scores.

**Conclusions:**

This study describes for the first time the involvement of white line disease, interdigital phlegmona, digital dermatitis and interdigital hyperplasia in foot disorders of dairy Mediterranean buffalo and shows their association with an impairment of animal welfare.

## Background

Foot health has been classified as the most important welfare problem in dairy cows, and its surveillance is the most representative animal-based indicator of welfare in dairy cattle [1, 2]. Additionally, lameness due to foot disorders represents the third most important health-related cause of economic loss in dairy industry after reproduction disorders and mastitis [3]. The financial loss can be measured in terms of decreased milk yield [4], reduced reproductive performance [5], increased culling rate, and increased production costs [4].

In Europe, the number of Mediterranean Buffaloes (MB) bred is ~450.000 animals, of which ~84 % is from Italy [6]. Although, these ruminants and dairy cows share similar breeding systems predisposing to similar herd problems [7], only reproductive disorders [8] and mastitis [9, 10] have been recognized as cause of important economic loss in MB so far. Foot diseases were always considered virtually non-existent in MB herds [11]; as a consequence, only a few and incomplete studies exploring the role and economic relevance of foot disorders (FDs) in this dairy population have been performed [11, 12].

As widely known in cows, FDs impair animal welfare and some of the most important are associated with lameness [13], reduction in body condition score (BCS) [14], and impairment of cleanliness score (CS) [15]. Indeed in herd with high percentage of FDs, lameness and behavioral changes (e.g. reduced total eating time, meals per day, increased lie down time, etc.) of the affected animals are often observed [16, 17], as a consequence of discomfort and a painful status [18–20]. As in cows, a Welfare Quality® [21] system (www.welfarequality.net) was developed also for Mediterranean buffaloes; lameness, BCS and CS are some of the criteria and measures for welfare assessment introduced by this project [22, 23].

The most common FDs in dairy cows comprise: sole ulcers (SU), white line disease (WLD), digital dermatitis (DD) and interdigital necrobacillosis (interdigital phlegmon – IP) [24, 25], while in MB, claw overgrowth (OG) and formation of corkscrew claws (CC) are the only claw disorders described until now [26]; no data are available about other type of FDs causing lameness.

In the field, the diagnosis of FDs can sometimes be challenging, because not all lesions cause lameness, and animals often have more than one type of lesion per foot; SU and IP, when present, are likely to be the cause of lameness while WLD, DD and heel horn erosion can frequently be present in non-lame cows [13]. In these animals a remarkable clinical support could be provided by novel electronic sensor systems able to automatically detect lameness due to foot lesions, actually not available for MB [27–29]. Considering these premises, the aim of this study was to describe the clinical findings of foot disorders (FDs) observed during routine foot trimming in dairy MB and their influence on animal welfare.

## Methods

### Animals and farms

The survey was performed on 1297 MB from 4 free-stall dairy farms in the Latina District - Middle Italy. This samples size was calculated by using the formula proposed by Thrusfield [30] considering the following values: study population (378.000 MB head in 2015) [31], expected prevalence of FDs (39.5 % in multiparous cows) [32], confidence interval 95 % and desired absolute precision (5 %).

Farms were selected following convenient sampling by the local veterinary practicing claw-trimmer. All of them were characterized by barns with solid grooved concrete floors in the walking and feeding alleys. The lying area consisted either of elevated cubicles covered with rubber mattresses for milking MB or of a roofed deep straw yard area for dry MB. In the farms enrolled, similar cleaning procedures were employed. All the animals enrolled were fed with a total mixed ratio including hay, silage and a multi-vitamin integrator. In all farms, milking animals were fed and milked two times/day. All the enrolled farms were characterized by a spring-summer deseasonalized calving system.

### Data acquisition

All the procedures performed in this study followed the common *good clinical practices* and received an institutional approval by Ethical Animal Care and Use Committee of University of Naples “Federico II”; moreover the farmers were previously informed and in agreement with purpose and methods used. Dairy farms were visited between April 2014 and March 2015. Routine foot trimming procedures were performed on all multiparous buffalo cows (≥2 lactations), whether lame or not, by the same investigator, one-time per year.

### Foot disorders

Presence or absence of FDs, as well as their localization, were recorded at claw trimming. The lesions observed in buffalo cows were categorized according to the ICAR Claw Health Atlas for cows [33], because of the lack of previous studies describing FDs in buffaloes.

Similar to the definition in cows [34], claw overgrowth (hereafter referred to as “overgrowth” [OG]) in MB was considered as an excessive elongation of the claws. No studies describing claws’ anatomical differences between MB and cows were published so far. According to the experience of the authors, the normal dorsal wall length (distance from coronet to tip of claw) in MB (after the foot-trimming) is usually considered 9 cm. As a consequence, lengths ranging from ≥ 9.1 to ≤ 10 cm were considered as moderate OG, but still within an acceptable range; a dorsal wall length exceeding 10 cm was instead considered affected by severe OG and categorized as FD by the authors in the current study.

Regarding CC, as for cows, it was considered as any torsion of either the outer or inner claw characterized by presence of the dorsal edge of the wall deviating from a straight line [33]. Corkscrew claw was diagnosed, if complete correction of the torsion at claw trimming was not possible.

### Animal welfare

All the animals affected by FDs were submitted to a complete clinical examination and their welfare status was determined by assessment of locomotion score (LS), body condition score (BCS) and cleanliness score (CS).

#### Locomotion score

The LS of each animal was assessed and recorded before trimming by one and the same investigator to avoid inter-observer differences in scoring. Observations were performed from the side and the rear of the buffalo cow, while it was moved to the trimming chute on flat and non-slippery surfaces. Buffalo cows were evaluated for their lameness status using the 5-point LS system described by Sprecher et al. [35] based on observation of posture and gait. The score was also dichotomized in a group with lameness status “non-lame” (scores 1–2) and a group with lameness status “lame” (scores 3–5) for the statistical analysis. A mobility score system validated for cows was used in the present study due to the lack of a classification system specific for MB. A LS > 2 was considered indicative of impairment of welfare.

#### Body condition score

All the scorings were performed by one and the same investigator when the animals affected by FDs were confined in the trimming chute. The BCS was estimated by visual inspection of the animals, and manual palpation of four areas of the body where MB store fat: ribs, spine, hips and base of the tail (hereafter referred to as “tail”) according to a 5-point scoring method introduced by [36] and slightly modified by the authors (Table 1). A score within the range of 1 (emaciated) and 5 (obese), with increments of 0.25 was given to each animal enrolled (score 3 = ideal BCS). For the analysis, BCS was grouped as under-conditioned (BCS ≤ 2.50), normally conditioned (BCS ≥ 2.75 and ≤ 3.50), and over-conditioned (BCS ≥ 3.75). Impairment of welfare was considered in animals that were under or over-conditioned (BCS ≤ 2.5 or ≥3.75, respectively).

**Table 1:** Description of assessment criteria for individual components of the BCS in Mediterranean buffaloes (ribs, spine, hips, and tail) according to Ezenwa et al. [36] modified

Region of body				
Score	Ribs	Spine	Hips	Tail
5 (Obese)	Not visible; fatty layer on and between ribs	Spine bones not visible. Spine sits in slight depression between fatty bulges left and right of spine	Convex, smooth rear, hip bones not visually apparent	Tail base sits in depression surrounded by soft fatty tissue
4	Few ribs visible towards abdomen; ribs can be felt	Spine bones not visible. Spine feels flat; bone and surrounding tissue are on level	Hip bones can be seen, round smooth appearance and feel	Tail base on level with surrounding fatty tissue
3 (Ideal)	Some ribs visible in center of ribcage; abdominal ribs feel ridged	Spine palpable as a slightly elevated bone center-line	Points of hips distinctly visible; bone easy to feel but not protruding	Tail base protrudes slightly; obvious by touch, but not by sight
2	Ribs visible throughout; all have ridged feel	Individual spinal vertebrae clearly palpable	Points of hips protrude; flanks are concave	Tail base visibly sticks up from surrounding tissue
1 (Emaciated)	Ribs clearly visible with deep depressions between them; very ridged feel	Vertebrae distinguishable by sight and touch	Hip bones protrude beyond the hip point; emaciated rear	Tissue surrounding tail base forms round hollow defined by pelvis

#### Cleanliness score

Cleanliness score was assessed using a scoring method previously reported by Nielsen et al. [15] for cows. Briefly, hind feet and limbs were the anatomical area evaluated always by the same investigator before foot-trimming. The scoring system aimed to track the amount of manure present and the distance it extended proximally up the leg by means of an ordinal scale from 1 to 4, in which: 1 = little or no manure above the coronary band; 2 = minor splashing above the coronary band; 3 = distinct plaques of manure above the coronary band, but with hair coat visible; 4 = solid plaques of manure extending high up to the hock and stifle. To the knowledge of the authors, no studies describing the effects of CS on MB welfare were published so far; as a consequence, according to the experience of the authors, values of CS > 2 were considered indicative of impairment of welfare.

Data regarding type of FDs, LS, BCS and CS were recorded on a spreadsheet and stored before the end of the foot trimming procedure, using the ear tag number of each buffalo cow to unequivocally identify individual animals. Information regarding farm management (type of feeding system, birth seasonality, type of barn, number of milking/day) as well as productive status of the animals with FD (milking or dried-off MB) were obtained from the farmers using a standardized questionnaire.

### Statistical analysis

All parameters were analyzed by standard descriptive statistics, and data distribution was assessed using histograms, normal probability plots and Shapiro Wilk tests. Data were expressed as absolute numbers, percentages and averages. Prevalences (PR), motility and lameness indexes (MI and LI, respectively) were also calculated. PR = E_+_/(E_+_ + E_−_), where E_+_ represents the number of animals showing the “*Event*” studied, while E_−_ represents the number of animals without the *Event* but that can express it (animals at risk); MI = number of cows not lame/number of multiparous cows in the herd; LI = number of cows lame/number of multiparous cows in herd [37]. Significant differences between expected and observed frequencies of categorical data (type of FD, LS, BCS, CS and productive status) were assessed by means of contingency tables, using *χ*^2^-test. Probabilities of *P* < 0.05 were considered significant. Data were analyzed using dedicated software (SPSS, Version 17.0, Chicago, IL).

## Results

### Prevalence of foot disorders

The prevalence of animals affected by FDs was 17.7 % (229/1297): overgrowth (OG) was present in 17.0 % (220/1297) of the MB enrolled, corkscrew claw (CC) in 15.8 % (205/1297), interdigital phlegmon (IP) in 0.9 % (12/1297), white line abscess (WLA) in 0.8 % (11/1297), digital dermatitis (DD) in 0.1 % (1/1297) and interdigital hyperplasia (IH) in 0.1 % (1/1297). Considering the whole sampling population, simultaneous presence of FDs was recorded in 17.0 % of MB (221/1297): OG + CC occurred together in 15.8 % of cases (205/1297), OG + IP in 0.3 % (4/1297), OG + WLA in 0.8 % (11/1297) and DD + IH in 0.1 % (1/1297). Significant associations between OG and CC [*χ*^2^ = 1191.967, degree of freedom (df) = 1, *P* < 0.0001] and between OG and WLA (*χ*^2^ = 54.311, df = 1, *P* < 0.0001) were detected. No lameness caused by orthopedic pathology located outside the feet was detected during the investigation.

Prevalence, localizations and type of disorders in the population affected by FDs are indicated in Table 2. Briefly, considering the group of MB affected by a FD, 26.2 % (60/229) of FDs were located in the front limbs, while 73.8 % (169/229) were located in the hind limbs, and 7.8 % (16/205) both in front and hind limbs. Of all FDs identified, 216 (94.3 %) were non-infectious disorders and 13 (5.7 %) infectious disorders.

**Table 2:** Type, prevalence and localization of foot disorders recorded in Mediterranean buffaloes (*n* = 229)

FDs	Prevalence	Limb	Prevalence	Claw	Prevalence
OG	96.1 % (220)	FL + HL	100 % (220/220)	/	/
CC	89.5 % (205)	FL	27.8 % (57/205)	MC	100 % (57/57)
				LC	/
		HL	72.2 % (148/205)	MC	93.9 % (139/148)
				LC	6.1 % (9/148)
IP	5.2 % (12)	FL	8.3 % (1/12)	/	/
		HL	91.7 % (11/12)	/	/
WLA	4.8 % (11)	FL	27.3 % (3/11)	MC	100 % (3/3)
		HL	72,7 % (8/11)	LC	100 % (8/8)
DD	0.4 % (1)	HL	100 % (1/1)	/	/
IH	0.4 % (1)	HL	100 % (1/1)	/	/

(x) = number of buffaloes; *FD* foot disorder, *OG* overgrow, *CC* corkscrew, *IP* interdigital phlegmon, *WLA* white line abscess, *DD* digital dermatitis, *IH* interdigital hyperplasia, *FL* front limb, *HL* hind limb, *MC* medial claw, *LC* lateral claw

### Animal welfare

Of all buffaloes affected by FDs, 90.0 % (206/229) showed LS score > 2, 19.6 % (45/229) a BCS ≤ 2.5 and 59.8 % (137/229) a CS > 2.

#### Lameness prevalence and locomotion score

Motility and lameness indexes recorded in the overall sample population were 84.1 % (1091/1297), and 15.9 % (206/1297), respectively; 57.3 % (118/206) of lame MB were milking while the 42.7 % (88/206) were dried-off. considering all the animals enrolled, lameness was always associated with the presence of at least one FD (LS > 2) (*χ*^2^ = 1142.13, df = 1, *P* < 0.0001), except for 23 MB with simultaneous occurrence of OG + CC. Significant differences between expected and observed frequencies of various LS on buffaloes affected by FDs were found for OG (*χ*^2^ = 94.125, df = 3, *P* < 0.0001), CC (*χ*^2^ = 229.000, df = 3, *P* < 0.0001), IP (*χ*^2^ = 157.922, df = 3, *P* < 0.0001) and WLA (*χ*^2^ = 133.072, df = 3, *P* < 0.0001). Prevalence, type of FDs and associated degree of lameness in the 229 buffaloes with FDs are described in detail in Table 3. Briefly, 10.0 % (23/229) of MB were scored as LS2 (non-lame group), while 79.5 % (182/229), 7.4 % (17/229) and 3.1 % (7/229) as LS3, LS4 and LS5, respectively (lame group). No statistically significant difference was found between MB with different productive status (milking versus dried-off) concerning their lameness status (lame versus non-lame).

**Table 3:** Type and prevalence of foot disorders in lame and non-lame Mediterranean buffaloes (*n* = 229) associated with different locomotion scores

LS	OG + CC	OG + IP	OG + WLA	DD + IH	IP
2	10.0 % (23)	-	-	-	-
3	79.5 % (182)	-	-	-	-
4	-	1.7 % (4)	2.2 % (5)	-	3.5 % (8)
5	-	-	2.6 % (6)	0.4 % (1)	-

(x) = number of buffaloes, *LS* locomotion score, *FD* foot disorder, *BCS* body condition score, *CS* cleanliness score, *OG* overgrow, *CC* corkscrew, *IP* interdigital phlegmon, *WLA* white line abscess, *DD* digital dermatitis, *IH* interdigital hyperplasia

#### Body condition score

Regarding the BCS, none of the MB with FDs were over-conditioned (BCS ≥ 3.75), while 80.3 % (184/229) were normal-conditioned (BCS ≥ 2.75 and ≤ 3.5) and 19.7 % (45/229) were under-conditioned (BCS ≤ 2.5). No statistically significant difference was found in MB with FDs between lameness status lame (*n* = 206) versus non-lame (*n* = 23) concerning BCS distribution. Of all MB with FDs, such with OG or CC were mainly associated with a BCS ranging between 2.50 and 3.00 while MB with WLA or IP mainly showed a BCS of 2.25 and 2.5; the only case of DD + IH detected showed a BCS of 2.75. A significant association between BCS and OG (*χ*^2^ = 33.408, df = 3, *P* < 0.0001) as well as between BCS and CC (*χ*^2^ = 123.877, df = 3, *P* < 0.0001), BCS and IP (*χ*^2^ = 46.006, df = 3, *P* < 0.0001), BCS and WLA (*χ*^2^ = 206.966, df = 3, *P* < 0.0001) were detected.

Prevalence and type of lesions in lame and non-lame MB enrolled affected by FDs, according to BCS observed, are reported in Table 4.

**Table 4:** Type and prevalence of foot disorders in lame and non-lame Mediterranean buffaloes associated with different body condition scores and cleanliness scores

Groups	BCS	FD	Prevalence	LS
Non-lame (23)	2.50	OG + CC	8.7 % (2)	2
	2.75	OG + CC	65.2 % (15)	
	3.00	OG + CC	26.1 % (6)	
Lame (206)	2.25	OG + IP	0.5 % (1)	4
		OG + WLA	2.4 % (5)	
		OG + WLA	1.9 % (4)	5
	2.50	OG + CC	10.2 % (21)	3
		IP	3.9 % (8)	4
		OG + IP	1.0 % (2)	
		OG + WLA	1.0 % (2)	5
	2.75	OG + CC	75.7 % (156)	3
		OG + IP	0.5 % (1)	4
		DD + IH	0.5 % (1)	5
	3.00	OG + CC	2.4 % (5)	3
Group	CS	FD	Prevalence	LS
Non-lame (23)	1	OG + CC	30.4 % (7)	2
	2	OG + CC	4.3 % (1)	
	3	OG + CC	8.7 % (2)	
	4	OG + CC	56.5 % (13)	
Lame (206)	1	OG + CC	34.0 % (70)	3
	2	OG + CC	6.3 % (13)	3
		IP	0.4 % (1)	4
	3	OG + CC	8.2 % (17)	3
		OG + IP	0.5 % (1)	4
		OG + WLA	0.5 % (1)	
		OG + WLA	0.5 % (1)	5
	4	OG + CC	40.0 % (82)	3
		OG + IP	1.5 % (3)	4
		IP	3.4 % (7)	
		OG + WLA	1.9 % (4)	
		OG + WLA	2.4 % (5)	5
		DD + IH	0.5 % (1)	

(x) = number of buffaloes, *LS* locomotion score, *FD* foot disorder, *BCS* body condition score, *CS* cleanliness score, *OG* overgrow, *CC* corkscrew, *IP* interdigital phlegmon, *WLA* white line abscess, *DD* digital dermatitis, *IH* interdigital hyperplasia

#### Cleanliness score

The CS 4 was recorded in 50.2 % (115/229) of MB affected with FDs, while it was 3 in 9.6 % (22/229), 2 in 6.6 % (15/229) and 1 in 33.6 % (77/229), respectively. In the MB with FD (lame and non-lame), the CS ranged between 1 and 4. Overgrowth and CC were detected at any scoring point, WLA and IP mainly at scores 3 and 4, and the only case of HH + ID showed score 4. Significant associations were found between CS and CC (*χ*^2^ = 15.335, df = 3, *P* < 0.01) as well as between CS and WLA (*χ*^2^ = 7.824, df = 3, *P* < 0.05). No associations were instead recorded for CS and OG (*χ*^2^ = 7.135, df = 3, *P =* 0.068), and CS and IP (*χ*^2^ = 7.106, df = 3, *P =* 0.069).

In Table 4, prevalence and types of lesions are given in lame and non-lame buffaloes enrolled, according to CS observed.

## Discussion

In this study, the clinical findings of foot disorders (FDs) affecting animal welfare are outlined for the first time in Mediterranean Buffaloes. Some FDs reported were never observed before in these ruminants. As a consequence, a comparative analysis with cows was done; in this context, the sample size was reached using the prevalence data originating from similar studies in cows [32], and it was more than tripled to increase the data’s reliability.

The overall lameness index detected in the 1297 MB enrolled was 15.9 % (206/1297); similar results were found in analogous studies performed in dairy cow farms in several European countries. Indeed in recent studies, the prevalence of lameness in dairy cows ranged from 14.8 to 19.0 % [24, 38]. Farmers, farm staff as well as veterinary practitioners traditionally tended to underestimate lameness in MB for a long time. This may be due to the low level of expression of the signs of pain by MB on one hand, and the lack of serious investigations performed in this field on the other hand. As described for cows, farmers may also do not feel the real necessity to investigate the problem, since they became tolerant to the levels of lameness in their herd, because the situation was similar to that of other herds they know [39]. Furthermore, unlike mastitis and infertility, from the farmers’ perspective, lameness does not overtly impact on farm economics, as lame animals continue to produce milk. So, for busy farmers to care for a lame animal is of low priority [39]. In the current study, the analysis did not reveal a significant difference of lameness prevalence according to the productive status; however it is necessary to highlight that the lack of more accurate data regarding the exact phase of lactation (e.g.: fresh MB or early-, mid-, or late-lactation animals) or dry-off (e.g.: dry or close-up MB) and milk yield may represent one of the restrictions of this study.

Foot disorders observed were mainly localized on the hind feet (73.8 %) and less frequently on the front feet (26.2 %), confirming similar results recently described by Somers and O’Grady [25] for dairy cows, where 11.8 and 89.6 % of the cows showed lesions recorded on front and hind feet, respectively.

Claw overgrowth (OG) represented the most frequent FD found in this study (96.1 %–220/229). Size and shape of the claw at any one time is a balance between growth and wear rate; several factors such as age of the animals, high concentrate feeding, degree of exercise and surface characteristics of the paddock and the stall alleys significantly influence the horn growth [40]. As shown in Table 3, severe OG was always detected in association with foot diseases (CC, WLA and IP) and the most frequent combination observed was represented by OG + CC (205/229). As widely described for cows [34], claw OG predisposes for the occurrence of others FDs potentially causing lameness also in MB.

During the current investigation, claw corkscrew (CC) was the most common non-infectious foot disease recorded both in the lame (182/206) and non-lame (23/23) group of MB (Tables 2 and 3), and it was observed only associated with OG as reported also by Cammarano and Marino [26]. In MB such as in cows, CC has been mainly ascribed to genetic heritable disorders, transmitted by bulls carrying the defects which should basically be excluded from reproduction [12, 40].

Regarding the influences of these two FDs on lameness, our data suggest that both OG and CC, when not associated with other foot diseases, are responsible for a mild (L2) to moderate (L3) lameness (*P* < 0.0001) (Table 3); the result obtained is in contrast with Napolitano et al. [11], who indicated this clinical sign as virtually absent in the 3 buffalo farms examined. Nevertheless, the LS values observed did not significantly influence the other welfare parameters considered (BCS and CS, Table 4), showing that these two FDs may not substantially affect buffaloes’ welfare.

Regarding the white line abscess (WLA), it represents a white line disorder mainly secondary to weakness of the white line, resulting, at the same time, in an abnormal horn production as a consequence of a laminitic insult (coriosis) [34]. White line disorders are considered some of the most important causes of severe lameness in cows [32]. While 4.8 % of the MB with FDs showed signs of WLA (Table 2), the values observed were lower than those recently described for lame cows, where the prevalence of WLA ranged from 17.2 to 23.5 % [25, 32].

In the present investigation, WLA was significantly associated with OG (*P <* 0.0001, Table 3); in agreement with Blowey [40], also in MB claw overgrowth could be considered as predisposing factor for white line disorders, increasing significantly the weakness of the hoof. Furthermore, as reported by Barker et al. [41] in cows, several factors can significantly increase the risk of WLA occurrence such as herd size, access to pasture and solid grooved concrete floors; in particular shallow grooves or loss of friction of solid grooved concrete over time lead to slippery floor surfaces associated with an increase in lameness, alterations in gait and increasing pressure on the sole and white line [42]. Solid grooved concrete floors were present in the alleys of all the farms enrolled and may have contributed to the appearance of the FDs, although this hypothesis lacks further scientific evidence. As proven by Whay et al. [19], white line disorders can also reduce cow’s nociceptive threshold increasing the sensitivity to pain. As shown in Tables 3 and 4, WLA was always associated with severe clinical lameness, suggesting that the pathology is painful also in MB (*P* < 0.0001, LS ≥ 4). As a consequence, the stressful condition experienced by the MB affected by WLA seemed to significantly influence the other welfare parameters considered: all of the animals were indeed in the under-conditioned group (BCS ≤ 2.5–11/11; Table 4) and showed values of cleanliness score above the threshold (CS3: 2/11–CS4: 9/11; Table 4). Similarly to severely lame cows, the authors suppose that, also in MB, this impairment of welfare might mainly be the consequence of less active animals, reduced total eating time, numbers of meals per day or dry matter intake as well as a longer lying time [16, 43]; further studies are necessary to confirm this hypothesis.

Interdigital phlegmon (IP) represents the second most frequent foot infectious disease observed in the group of lame MB (5.2 %, Table 2); a similar prevalence (4.4 %) was described by DeFrain et al. [32] in cattle. Injuries of the interdigital skin are usually a prerequisite for the infection; stones, stubble, kale stems, hardened dung in the interdigital area, sticks, very dry pasture, or rough flooring can easily damage the area of the feet involved [34, 40]. A bacterial invasion of the skin of the interdigital cleft mainly due to *Fusobacterium necrophorum* is usually associated with IP in cattle [39]. Unfortunately, no information regarding the injuries promoting the infection and the type of microorganisms primarily involved in MB’ IP are available, representing another restriction of the study.

As described by Whay et al. [18, 19], lesions of the interdigital skin are often related to hyperalgesia and consequently to lameness. This seems to hold true also in MB, because all the animals affected by IP had a severe lameness with gaits showing hesitating steps (*P* < 0.0001) (LS4, Table 3). Moreover, considering the effects on BCS and CS, 91.7 % (11/12) were in the under-conditioned group (BCS ≤ 2.75, *P* < 0.0001) and rated with CS > 2 (CS3: 1/11–CS4: 10/11, *P* = 0.068, Table 4). These results showed a lower effect of IP on impairment of behavior and welfare of affected animals than WLA.

Regarding digital dermatitis (DD), a single case of a DD-associated interdigital hyperplasia (IH) was detected in the present study. Digital dermatitis is known as bacterial skin infection primarily affecting the epidermis and seriously compromising the welfare and productivity of affected cattle [44]. It was described for the first time in Italy by Cheli and Mortellaro [45] and since then, it rapidly spread all over the world. The DD lesion occurred on the tip of IH and was diagnosed according to the clinical picture, showing close similarity to that in dairy cattle. Neither histological evaluation nor PCR for the relevant *Treponema* species were performed. In comparison to the situation in dairy cattle [24, 25, 32], the prevalence of DD was very low in MB, although management of both dairy species is very similar. The lack of a previous description in MB could be both related to the inability to recognize DD (if associated with a low LS) as well as to a lower sensitivity of this species to slurry exposure, commonly considered a major risk factor of DD in dairy cows [46]. This clinical findings merit further scientific attention.

As last, it is interesting to underline that other noninfectious foot diseases, such as sole ulcers (SU), were not found although they occur frequently in dairy cows. The pathogenesis of SU in dairy cow was recognized as the consequence of abnormal compression of the corium and nutritional imbalances, leading to ischemic necrosis, altered horn formation and formation of horn of poor quality, finally reaching the bearing surface of the sole [40]. The reason for the obvious difference in the prevalence of SU between MB and dairy cows is not known.

Making an overall judgement on the effect of FDs on welfare, as described for cows by Tadich et al. [13], a significant association between the presence of FDs and various degrees of lameness has been detected also in MB (*P* < 0.0001). The majority of MB within the under-conditioned group (95.5 %, 43/45) and all those with CS > 2 (122/122) had a locomotion score above the threshold of normality (LS > 2), adversely affecting buffaloes’ welfare. Furthermore, foot diseases such as IP, WLA and DD or IH but not CC and OG seemed to occur more frequently associated with decreased BCS and increased CS scores (Table 4).

## Conclusions

This study represents the first clinical description of involvement of white line disease, interdigital phlegmona, digital dermatitis and interdigital hyperplasia in foot disorders of dairy Mediterranean buffalo, revealing impairment of animal welfare of affected animals. More complete studies including the assessment of the pathogenesis, risk factors and economic impact of FDs on MB dairy industry are warranted. By improving the scientific knowledge concerning FDs of MB, awareness of the problem and empathy with the welfare of these dairy ruminants can be increased.

## Abbreviations

BCS, body condition score; CC, corkscrew; CS, cleanliness score; DD, digital dermatitis; FD, foot disorder; IH, interdigital hyperplasia; IP, interdigital phlegmon; LI, lameness indexes; LS, locomotion score; MB, Mediterranean buffaloes; MI, motility index; OG, overgrow; PR, prevalence; WLA, white line abscess
